# Prediction of effective genome size in metagenomic samples

**DOI:** 10.1186/gb-2007-8-1-r10

**Published:** 2007-01-15

**Authors:** Jeroen Raes, Jan O Korbel, Martin J Lercher, Christian von Mering, Peer Bork

**Affiliations:** 1 European Molecular Biology Laboratory, Meyerhofstrasse 1, D-69117 Heidelberg, Germany; 2 Molecular Biophysics & Biochemistry Department, Yale University, Whitney Avenue, New Haven, Connecticut, USA; 3 Institute of Molecular Biology, University of Zurich, Winterthurerstrasse 190, 8057 Zurich, Switzerland

## Abstract

We introduce a novel computational approach to predict effective genome size (EGS; a measure that includes multiple plasmid copies, inserted sequences, and associated phages and viruses) from short sequencing reads of environmental genomics (or metagenomics) projects. We observe considerable EGS differences between environments and link this with ecologic complexity as well as species composition (for instance, the presence of eukaryotes). For example, we estimate EGS in a complex, organism-dense farm soil sample at about 6.3 megabases (Mb) whereas that of the bacteria therein is only 4.7 Mb; for bacteria in a nutrient-poor, organism-sparse ocean surface water sample, EGS is as low as 1.6 Mb. The method also permits evaluation of completion status and assembly bias in single-genome sequencing projects.

## Abstract 2

A novel computational approach shows a link between genome size and habitat from analysis of environmental metagenomic DNA reads.

## Background

Because of its direct link with the functional repertoire, microbial genome size is an important ecologic parameter that is believed to be closely coupled to the functional complexity and environmental niche of an organism [1-4]. For more than three decades, numerous studies have provided estimates of average microbial genome size for various environments, but results vary greatly. Estimates of the average DNA content per cell (converted to megabases [Mb] for comparison) range from 1.5 to 8.0 Mb for soil and from 1.5 to 9.5 Mb for aquatic environments (see Loferer-Krossbacher and coworkers [5] and Torsvik [6] for overviews of estimates). However, the diversity of techniques and parameters used (for example, sample filtering, DNA staining, and cell counting) greatly hampers the interpretation and comparison of these results. All currently used methods also have several important drawbacks. For instance, they have difficulties discriminating between the different ploidy levels of cells [7-9], and so any technique measuring DNA content does not necessarily measure genome size. In addition, DNA binding of stains used in the majority of these studies (for example, DAPI) is not always specific, and important biasing factors (GC content, permeability, salinity, influence of debris, and so on) have hardly been compensated for [7,10-12]. Finally, some estimates have been obtained in studies using cultured isolates only (for example, se the reports by Christensen [8] and Torsvik [13] and their groups), which does not reflect the actual environmental species composition.

Because of all of these difficulties, the average genome size of micro-organisms living in particular environments is still uncertain, and the influence of environments on genome size remains a matter of speculation. Recently, however, several studies have provided unprecedented insights into the microbial DNA content of complete ecosystems using massive random shotgun sequencing of environmental samples [14-16]. Through comparative metagenomics, various aspects of ecologic complexity can now be studied [15,17-20]. Here, we use these data to study the relationship between environment and microbial genome size.

## Results and discussion

### The concept of effective genome size

An assembled, sequenced genome is a nonredundant representation of the naturally occurring amount of base pairs that a cell supports. It reflects neither actual copy number of inserted elements and plasmids nor the amount of associated phages and viruses. However, the total amount of DNA replicated per cell division is what determines the metabolic cost and what has to be balanced against the full functional spectrum of genes available to a given organism. To estimate the latter, ecologically more meaningful measure of genome size (subsequently referred to as 'effective genome size' [EGS]), we have developed a novel computational approach to predict EGS directly from raw shotgun sequencing data, thereby avoiding experimental biases such as are mentioned above. When applied to metagenomics data, our method measures the average EGS of organisms living in the sampled environment.

### Deriving a method for EGS prediction

In brief, we use a set of marker genes that typically occur only once per genome to extrapolate the average genome size from the density of these genes found in the total set of sequence reads. Even in complex metagenomics data, the total number of marker genes should be proportional to the number of genome equivalents (and thus individuals) present, and the marker gene density (number of marker genes divided by the number of sequenced base pairs) should thus be inversely correlated to the average size of the genomes in the sample. This approach would also be able to normalize, unlike previous DNA measurements, for intermittent episodes of polyploidy (for example, in the case of fast-growing microbes that may replicate multiple concurrent copies of their genomes); in these situations our marker genes themselves are present in multiple copies and their density does not change.

**Figure 1:**
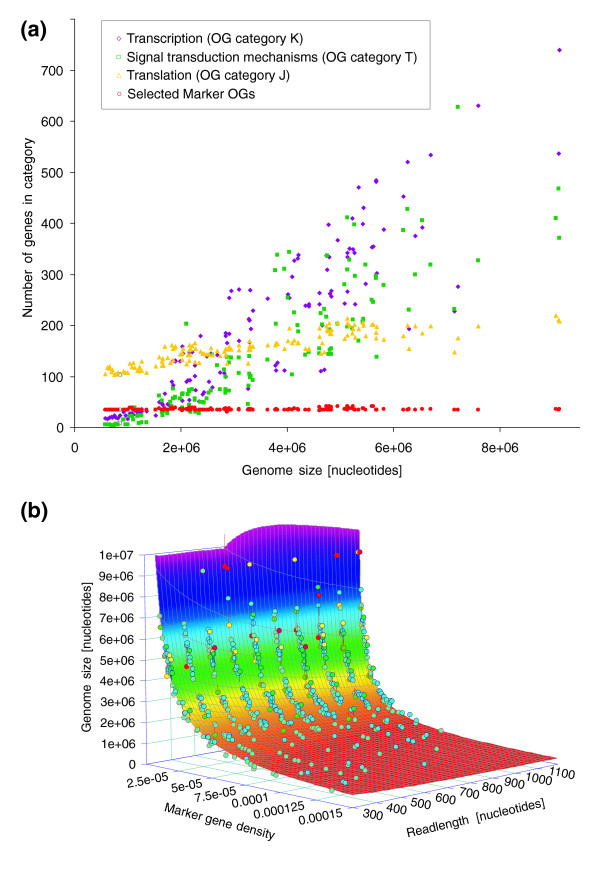
Predicting effective genome size from marker gene density. **(a) **Gene counts for various functional classes [50] and their relationship with genome size. Although counts of genes belonging to the categories T (signal transduction mechanisms), K (transcription) and J (translation, ribosome structure, and biogenesis) scale (to a greater or lesser extent) with genome size, the set of 35 universal, single-copy genes used in this study does not. **(b) **Calibration plane used to identify the relationship between marker gene density, read length, and genome size. The calibration was based on a simulated shotgun dataset of randomly extracted 'reads' from the sequenced genomes (see Materials and methods), because insufficient raw shotgun sequence data are currently available in the trace archives to allow a robust calibration based on 'real' data. Circles represent shotgun datasets. Circle fill color indicates the goodness of fit to the plane (blue = <1 standard deviation [SD], green = <2 SD, yellow = <3 SD, red = >3 SD). Circle border indicates position relative to plane (blue = above, red = below). OG, orthologous group.

Previous studies have shown that genes involved in translation, ribosome structure, and biogenesis generally exhibit a low number of duplicates per genome and their number does not expand much with genome size [2,21-24], and thus would constitute suitable marker genes to estimate the number of individuals in a sample, irrespective of genome size. However, when applying orthologous group (OG) categories for identifying such genes, we still observed a slight positive correlation between genome size and the number of translation-related genes (Figure 1a). Therefore, we selected a universally occurring set of marker genes (largely overlapping with the ones used by Ciccarelli and coworkers [25]) that only very rarely occur as duplicates, such that the total marker gene count remains constant with increasing genome size (Figure 1a; also see Materials and methods, below). The selected marker genes (most of them, but not all involved in translation) can be considered to be both essential for cellular life and very ancient; they evolve at a slow rate and are members of basal cellular processes, exhibiting little variation across phyla.

To identify the relationship between the density (count per megabase [Mb]) of the combined set of these selected markers and genome size, we calibrated our method on simulated shotgun reads from 154 completely sequenced bacterial and archaeal genomes (see Materials and methods, below). Indeed, although the relationship between the true number of occurrences of each marker gene and the number of individuals (and thus the relationship between their density and average genome size) is simple, the relationship between the number of BLAST-observable instances of a combined set of marker genes in incomplete environmental shotgun data and the number of individuals is not so straightforward. In addition, the length of sequencing reads can seriously influence the likelihood of successfully detecting a marker gene using BLAST (data not shown). Therefore, we performed a three-dimensional calibration relating marker gene density *x*, read length *L*, and genome size (Figure 1b; see Materials and methods, below), resulting in the following relationship:

EGS=a+b×L−cx
 MathType@MTEF@5@5@+=feaafiart1ev1aaatCvAUfeBSjuyZL2yd9gzLbvyNv2Caerbhv2BYDwAHbqedmvETj2BSbqee0evGueE0jxyaibaiKI8=vI8tuQ8FMI8Gi=hEeeu0xXdbba9frFj0=OqFfea0dXdd9vqai=hGuQ8kuc9pgc9s8qqaq=dirpe0xb9q8qiLsFr0=vr0=vr0dc8meaabaqaciGacaGaaeqabaqadeqadaaakeaacaWGfbGaam4raiaadofacqGH9aqpdaWcaaqaaiaadggacqGHRaWkcaWGIbGaey41aqRaamitamaaCaaaleqabaGaeyOeI0Iaam4yaaaaaOqaaiaadIhaaaaaaa@3F51@ (with *a *= 21.2, *b *= 4230, and *c *= 0.733)

This relation can be used to predict genome size (in Mb) from the two other parameters *x *and *L*. This formula indeed shows the inverse relationship between EGS and marker gene density, but corrected by a read-length-dependent factor following a power law. An analysis of sources and magnitudes of errors in predicted genome sizes showed that inaccuracies stem mostly from finite sequencing depth, from uncertainties associated with identification of marker gene sequences using BLAST, and from residual biological variation in genomic marker gene count (see Additional data file 1). The error contribution from finite depth is small when more than about four times the average genome size was sequenced (Additional data file 2; this is the case in the environmental shotgun-sequenced samples currently available). On the whole, the median unsigned prediction error on the simulated shotgun data was 5.3% (standard deviation [SD] 8.7%), largely independent of genome size and read length (Additional data file 2).

Because marker genes are equally present in all species, our method should work well on complex, mixed samples (a theoretical proof can be found in the Additional data file 1). We further support this by performing simulations mixing species and read lengths. For mixtures, the contribution of each species should weighted by the fraction (in numbers of genomes) it takes up in the sample. For example, for a mixture of two species of 4 Mb (90% of genomes) and 12 Mb (10% of genomes), we should get EGS = 0.9 × 4 Mb + 0.1 × 12 Mb. Our simulations show that this is indeed this case (see Materials and methods, below; Additional data file 1; and Additional data file 3).

### Method validation on real shotgun data and detection of sequencing artifacts

**Figure 2:**
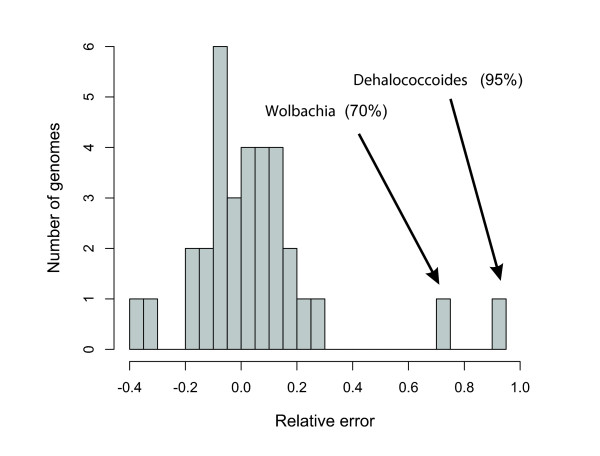
Prediction error and identification of sequencing artifacts. Distribution of the prediction error ([predicted - known genome size]/known genome size) of 32 complete genome shotgun datasets downloaded from the NCBI's trace archive (see Additional data file 9 for a list). The majority of predictions have an error estimate <20%, with a median value of about 9%. There are, however, two exceptions in which the error is significantly larger. The first is the *Wolbachia *endosymbiont of *Drosophila melanogaster*. The marker OG density in the simulated reads is considerably higher than in the real shotgun data, leading to a 70% difference in predicted genome size. After further investigation of the raw reads, we noticed that this difference was caused by an important contamination of the dataset by reads originating from the organism's host, *Drosophila*, that were filtered out during the assembly of the genome but that are still present in the shotgun data available at the trace archive. The second exception is the genome of the PCE-dechlorination bacterium *Dehalococcoides ethenogenes*. Also here, the marker OG density in the shotgun data is lower than in the simulated dataset. Mapping of the publicly available reads to the genome sequence showed a peak of read density in a region that was identified to be an integrated element that is believed to exist in variable copy numbers in different individuals but was only included once in the published genome sequence [51]. OG, orthologous group.

To confirm that the simulated data represent a valid approximation of real sequencing reads, we measured the prediction error on publicly available sets of microbial whole-genome raw shotgun sequencing reads (Figure 2). (Note that only 32 such datasets were present in NCBI's trace archives at the time of analysis; a larger training set of whole-genome sequencing reads should allow further improvement of the method.) The analysis of this prediction error on 'real' reads revealed a systematic shift of predicted versus known genome sizes by 15.9%, most likely reflecting unequal representation of certain genomic regions in sequencing libraries ('cloning bias' due to library preparation, toxicity, restriction site biases, and other factors). After correction for this bias (which leads to an adjustment of the values for *a *and *b *in Eqn 1 [see Materials and methods, below]), the median error on real shotgun data is as low as about 7.8% (SD 14.4%; Figure 2; also see Materials and methods, below). The two outliers with larger errors can be linked to anomalies in the deposited reads, caused by contamination (*Wolbachia*) or collapsing of repeated insert sequences (*Dehalococcoides*; Figure 2). The latter example illustrates that the EGS predicted by our method does not just reflect the principal, nonredundant chromosome, but indeed it also considers the actual copy number of inserted elements and plasmids. The importance of this additional genomic repertoire ('mobilome' [26]) is not to be underestimated. For example, a 20% variation in chromosome length was described for different isolates of *Escherichia coli *[27].

Because these cases indicate that our approach can identify assembly artifacts or incomplete cloning material, we applied the method to unfinished genome sequencing projects that have not recently been updated and might have had a problematic project history (Additional data file 4). Our method appears to yield plausible results on the whole, as in the majority of cases our predictions stay within the error ranges of previous estimates based on, for example, pulse-field gel electrophoresis. In a few cases our method reveals a larger genome size than was initially anticipated, which might explain problems in achieving sufficient sequence coverage (such as for the *Chloroflexus aurantiacus *genome); here, our predictions can provide guidance as to the amount of sequence data needed for genome completion. This is expected to be particularly useful in sequencing projects that utilize the recently developed low-cost sequencing techniques [28,29], which produce short reads and are thus more difficult to assemble.

### Characterizing prokaryotic genome sizes as comparable subsets of samples

In complex environmental samples, the proportion of eukaryotic DNA present may have a large impact on EGS measurements. Because of their disproportionally larger genome size, even a minor fraction of eukaryotes in the sample could inflate EGS. Thus, the relationship between organism complexity, EGS, and environment becomes difficult to interpret, even though eukaryotes are a valid part of an environmental sample and a higher proportion of eukaryotes should go hand in hand with a higher degree of complexity.

In order to better understand these effects, we adapted the method to measure EGS specifically for only the bacterial or archaeal fraction of the sample (see Materials and methods, below). To do this, we divide the number of hits to marker genes of bacterial/archaeal origin (as determined by the best BLAST hit in the STRING database [30]) by the number of hits to any bacterial/archaeal gene. Calibration of this domain-specific marker gene density on known genomes shows that, as expected, it scales inversely linear to genome size. The error is read-length-independent and is not influenced by genome size. Median prediction error for real bacterial shotgun reads is 8.0% (SD 14.6%) for the bacteria-specific measure (see Additional data file 1 and Additional data files 5 and 6). The archaea-specific measure is associated with a higher prediction error (see Materials and methods, below) because of the small number of genomes available for calibration and will improve when more genomes become available for this domain.

### Measuring effective genome size of real environments using metagenomics data

Having established methods for measuring EGS, we applied them to 12 publicly available environmental sequence data sets: five communities that were sampled without particle size filtering (a soil community, an acidophilic underground biofilm ['acid mine drainage'], and three deep sea whale carcass scavenger communities ['whale falls'] [14,15]), and also seven Sargasso Sea water samples of different cell size fractions. (For the latter, sea water was pumped through two consecutive filters: a first 'prefilter' to remove larger organisms and debris, and a second 'collection filter' for sampling. Therefore, each Sargasso Sea sample should be interpreted with the corresponding organismal size range in mind [16,31].)

**Figure 3:**
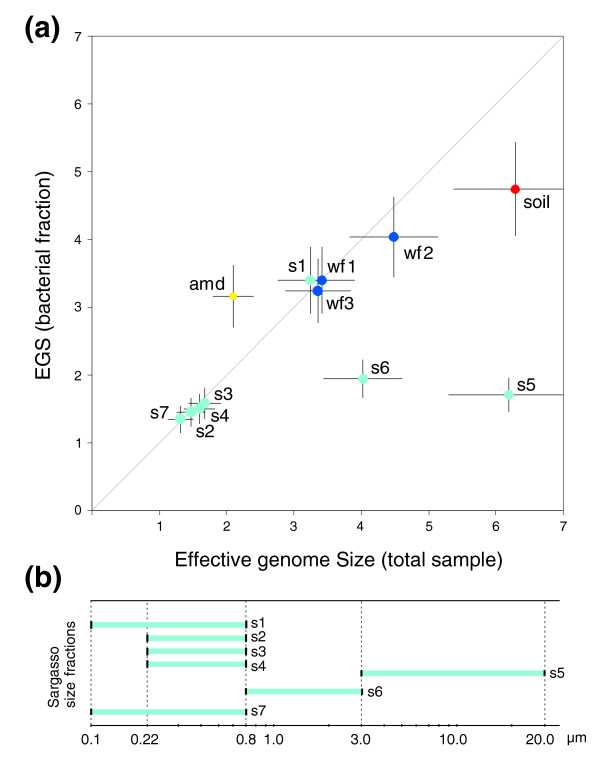
Predicted effective genome sizes for environments. **(a) **Comparison of predicted EGS for total samples versus the bacterial fraction. amd, acid mine drainage; wf, whale fall deep sea samples; s, Sargasso Sea samples. Error bars indicate standard deviation for total (horizontal) and bacteria-specific (vertical) estimates. **(b) **Overview of cell size in the different Sargasso Sea samples due to filtering during sampling.

Measurements of the EGS in the samples shows that the soil sample has the largest EGS, together with two of the Sargasso Sea samples of large cell size fractions, whereas the other Sargasso Sea samples have very low EGS estimates (Table 1). In order to test whether these values reflect functional complexity of the micro-organisms in the sample or only reflect phylogenetic composition of the samples, we applied our method of measuring bacterial/archaeal EGS. The comparison of the latter with the general EGS measure shows that in a number of the samples analyzed here, the presence of eukaryotes indeed has an important effect on the estimated EGS. For example, the value for soil is reduced by about 25% when eukaryotes are excluded. However, the most drastic differences are seen in the Sargasso Sea samples 5 and 6 (the two largest cell size fractions), which are reduced by more than 75% and 50%, respectively, now causing all Sargasso size fractions to become statistically indistinguishable and converge to an average bacteria-specific EGS of 1.6 Mb (sample 1 excluded [see below]; Table 1 and Figure 3). In addition, the fact that the samples originate from four different sampling sites in the Sargasso Sea (stations 11/13, 3, 13, and S) and were taken at different time points [16] suggests that microenvironmental differences are not influencing genome size or, alternatively, that there are very little differences in the bacterial populations of these sites. Indeed, water currents can allow for a rapid and continuous homogenization of communities, and previous studies showed very little variation in GC content between samples with similar filtering treatment [32], arguing for the latter explanation.

The only outlier in these estimates is that of sample 1 (station 13/11), which has a bacterial-specific EGS of 3.4 Mb, which is about twice as large as all other samples (Table 1). However, for this sample, contamination with *Shewanella *and *Burkholderia*, two terrestrial species, has been proposed [20,33], which could explain the EGS differences.

The acid mine drainage dataset derived from a biofilm at pH 0.83 [14] provides a first large-scale glimpse of genomic properties of free-living extremophiles. When only considering the bacterial EGS for the acid mine drainage sample, our results show an increase of 50% compared with the overall measure (3.2 Mb versus 2.1 Mb; Table 1 and Figure 3). This might be explained by the presence of small genome archaea (*Ferroplasma acidarmanus fer1 and Ferroplasma type II*), which (as estimated by BLAST-based phylotyping; data not shown) appear to dominate the deposited reads. Indeed, the calculated EGS of archaea in this sample (1.8 Mb) is in accordance with the genome sizes of the two assembled species. Intriguingly, the bacteria-specific EGS in the acid mine drainage sample (3.2 Mb) is more than twice as large as the average parasite/symbiont genome size [34] and is thus in conflict with the proposed theory that genome evolution patterns of free-living extremophiles are similar to those of intracellular pathogens or symbionts [35].

### Effective genome size correlates with environmental complexity

Although the Sargasso Sea samples are separated into size fractions, the convergence of all samples to a narrow common average bacterial EGS of about 1.6 Mb suggests that this value gives a correct general EGS of bacteria living in this environment. When considering only the bacterial fraction, we can hence compare these samples with the other (unfiltered) samples in order to investigate the influence of environmental factors on genome size. Our results show that soil bacterial EGS is significantly larger than that of the pooled noncontaminated Sargasso Sea samples (*P *= 5.5 × 10^-6^, after correction for multiple comparisons) and marginally significantly larger than acid mine drainage (*P *= 0.04) and the pooled whale falls (*P *= 0.053). Although the genomes of fully sequenced soil dwellers were already noted to have a tendency to be larger than others [1,21], we provide here - for the first time - conclusive evidence for this hypothesis based on an unbiased sampling of thousands of soil bacteria. Soil is a very challenging environment for two reasons: the high organism density, leading to strong competition for nutrients as well as complex communication and cooperation strategies; and the highly variable living conditions (for example, seasons and weather) [36]. Therefore, a broad functional repertoire is needed in order to survive competition and adapt to ever-changing conditions. Together with the fact that only about 50% of the predicted soil genes have a match in current protein databases, our results imply a wide variety of novel functions and processes in soil, potentially including biotechnologically relevant ones such as defense (antibiotics), biosynthesis, and biodegradation.

The EGS of the Sargasso Sea samples is significantly smaller than that of all other samples. The explanation could lie in the lower organism density in Sargasso surface waters (about three orders of magnitude smaller than soil [16,37]), which would allow organisms to shed the functional repertoire presumably needed for survival in densely populated, substrate-bound habitats, or alternatively 'genome streamlining' to optimize replication under limiting nutrient resources, as was seen for *Pelagibacter ubique *[34], a member of the SAR11 clade, and *Prochlorococcus *[38-40], which dominates oceanic surface waters. Our estimated EGS is consistent with the genome sizes of these organisms (1.3 Mb for *Pelagibacter *and 1.6 Mb for the *Prochlorococcus *high-light-adapted ecotype [34,39]) and the previously reported low GC content of Sargasso sequences [32], as GC content scales with genome size [1].

As expected, acid mine drainage and whalefall EGS estimates are in between both extremes, in accordance with their densely populated substrate-bound lifestyle, but under relatively stable environmental conditions [14,15].

## Conclusion

Using a novel computational approach, we have shown how EGS can be directly determined from raw sequencing reads, either for single species or entire organismal communities. EGS can be reliably estimated for complex environments as a whole, but also for bacterial or archaeal subcommunities in a sample. Applying this method to diverse environmental datasets, we could establish a relationship between genome size and environment, suggesting a clear correlation between environmental complexity and the diversity of the cellular repertoire that is required to cope with various external challenges. Because EGS directly reflects the functional diversity of a community, it will not only serve as a useful ecologic parameter but might also play a role in the search for novel biologic activities.

Furthermore, an accurate estimate of average genome sizes for different environments is paramount for other approaches to elucidating the totality of ecosystem composition and functioning. For example, widely used techniques such as DNA reassociation kinetics help one to understand ecosystem species composition and biodiversity as a whole [37,41], but they require knowledge of the average genome size to translate genetic diversity into species diversity. Even environmental cell counts (used for various applications) heavily depend on the selection of a reference species with a genome size comparable with the sampled ecosystem average [42]. For the analysis of metagenomics data, the average genome size allows one to calculate early in a pipeline the amount of sequencing necessary for completion of the most dominant species [15], and is needed for the deduction of community structures from assembly data [43]. Currently, our method is limited to predicting only the average EGS, without describing the distribution of genome sizes within the sample. However, improvements in phylogenetic separation of metagenomic sequences should allow the adaptation of our method to predict genome size distributions in the future.

The applications above illustrate the importance of this parameter (together with the functional and phylogenetic characterization of samples) in the process of elucidating ecosystem properties from metagenomics data. The EGS, as predicted here, is thus applicable to a broad range of questions and techniques ranging from genomics via population genetics to ecology.

## Materials and methods

### Detection of marker genes

We used a set of 35 OGs that are widely conserved (present in most species), rarely occur as duplicate genes in known genomes, are not subject to horizontal gene transfer, and do not scale with genome size as marker genes (set largely overlapping with the one used by Ciccarelli and coworkers [25]; Figure 1 and Additional data file 7). Marker gene counts were carried out using an approach similar to the one described previously [15], based on comparisons with known proteins. In brief, DNA sequence reads (or, alternatively, randomly generated genome fragments) were searched against the extended database of proteins assigned to OGs in the latest STRING release (6.3) [30], using BLASTX [44], and an OG was called present when a hit matching one of its proteins occurred (with a BLAST score of at least 60 bits). Note that this procedure is largely independent on varying annotation qualities across genomes, and avoids biases owing to lower gene prediction quality on short sequences [17], such as the reads used in this study.

In order to avoid potential biases introduced by any uneven phylogenetic representation within the reference set of known proteins, all BLASTX matches exceeding an overall protein identity of 50% were discarded. This latter step is needed to avoid artefacts introduced by the occasional organism in the sample that happens to be closely related to a known organism in the BLAST database. For such an organism, even marker genes contained only partially on a read can be detectable by BLAST because of their high sequence identity to known genes. In contrast, most environmental genes have low sequence identity to known genes, and so fragmented marker genes often escape detection. Thus, without the above threshold, marker gene counts would be higher for well known organisms, biasing the results. (Note that the threshold does not select against well known organisms either; their genes will still generate hits with other organisms in the database, with identities below 50%, and will thus be counted like any other environmental marker gene.)

Query sequences were allowed to map to several OGs, provided these overlapped by no more than 50% of the shortest assignment. BLASTX was run using the BLOSUM62 matrix, and low-complexity filtering was enabled. Marker gene density *x *was then defined as the number of matches to reference genes, divided by the total number of megabases surveyed.

### Calibrating marker gene density with genome size, and genome size prediction

To determine the relationship between the occurrence of marker genes and genome size, we used fully sequenced genomes for calibration. We simulated the widely used whole genome shotgun (WGS) sequencing process by randomly extracting 'reads' of variable read length from 154 previously completely sequenced bacterial and archaeal genomes (EBI Genome Reviews release 17). In total, 50 genomes were randomly chosen per read length bin (300, 400, 500, 600, 700, 800, 900, 1000, 1100, 1200 base pairs [bp]), and reads were sampled until 3× coverage was achieved (see Additional data file 8 for a list of genomes). We did not distinguish between plasmid and chromosomal DNA (each DNA fragment of a completely sequenced genome was equally likely to be considered).

We determined the occurrences of marker genes among these 'reads' as described above. On these counts, we based a three-dimensional calibration, relating known genome size to the parameters read length and marker gene density. Because the total number of marker genes per genome does not vary with genome size, we expect that genome size increases proportionally to the inverse marker gene density 1/*x *at any given read length *L*: EGS = *c*(*L*)/*x*, where *c*(*L*) is a read-length-dependent calibration factor. The exact form of *c*(*L*) is determined not only by the read-length dependence of the probability of sequencing a portion of a marker gene, but also by the probability of identifying a read as a marker gene. Because the latter depends on sequence features of individual marker genes, it is not easily possible to specify the analytical form of *c*(*L*) *a priori*. Based on manual comparison of a variety of possible functional forms, we found that *c*(*L*) is well approximated by a power law, *c*(*L*) = *a *+ *b *× *L*^-*c*^. This is indeed the best (R^2 ^= 0.97) 'simple' three-parameter formula relating genome size to marker gene density and read length, as confirmed using the TableCurve3D v.4.0 package; a 'simple' equation is defined here as a three parameter equation consisting of a constant and two coefficients that multiply a function of *x *or *L*. This resulted in the prediction formula:

EGS=a+b×L−cx     (1)
 MathType@MTEF@5@5@+=feaafiart1ev1aaatCvAUfeBSjuyZL2yd9gzLbvyNv2Caerbhv2BYDwAHbqedmvETj2BSbqee0evGueE0jxyaibaiKI8=vI8tuQ8FMI8Gi=hEeeu0xXdbba9frFj0=OqFfea0dXdd9vqai=hGuQ8kuc9pgc9s8qqaq=dirpe0xb9q8qiLsFr0=vr0=vr0dc8meaabaqaciGacaGaaeqabaqadeqadaaakeaacaWGfbGaam4raiaadofacqGH9aqpdaWcaaqaaiaadggacqGHRaWkcaWGIbGaey41aqRaamitamaaCaaaleqabaGaeyOeI0Iaam4yaaaaaOqaaiaadIhaaaGaaCzcaiaaxMaadaqadaqaaiaaigdaaiaawIcacaGLPaaaaaa@42D9@

We estimated the parameters of this formula with a nonlinear least-squares fit, as implemented with the *nls *function in the R programming environment [45]. First, we randomly selected half of the species in the simulated data. Their complete sets of simulation results (marker gene densities *x *at specified read lengths *L*), together with the known genome sizes *z*, were used as calibration data (the remaining data were later used for detailed error estimation; see Additional data file 1 and Additional data file 2). Parameters *a*, *b*, and *c *were chosen such as to minimize the weighted sum of squares ([*a *+ *b *× *L*^-*c*^]/*x *- *z*)^2^/*z*. This led to the parameter estimates *a *= 21.2, *b *= 4230, and *c *= 0.733.

Because Eqn 1 is linear in the inverse marker gene density *x*^-1^, it can be directly applied to mixtures of genomes, which was supported by our simulations. In the case of species mixtures, the estimated mean EGS is the number of megabases per genome present in the sample (effective genome sizes of different species are weighted by their genome count, not by their contribution to the number of sequenced base pairs). Because Eqn 1 is further approximately linear in the inverse read length *L*^-1^, it can also be applied to sequence datasets with mixed read lengths. (For a full discussion and simulation of EGS prediction in mixtures, see Additional data file 1 and Additional data file 3).

So far, calibration was based on simulated reads from fully sequenced genomes, ignoring potential biases introduced, for instance, by cloning. To test our predictions on actual sequencing data, we downloaded and analyzed shotgun data of completed genomes from the NCBI and Ensembl trace repositories [46,47], excluding those projects for which sequencing coverage was low (<1.5×; Additional data file 9). In order to ensure consistency we applied a uniform quality clipping method to all 32 datasets, rather than using the provided coordinates of each sequencing centre (phred quality cut-off score of 15, using a perl script kindly provided by Jarrod Chapman [JGI]; clipped reads with fewer then 100 nucleotides remaining were discarded). We found that Eqn 1 overestimates genome size on average by 15.9%. This reflects a strong bias against the marker genes, probably resulting from the fact that these 35 OGs were chosen for their strongly conserved single-copy distribution across genomes, and hence introduction of additional genes into the cloning vector is often lethal. Removal of this bias by an additional scaling factor (excluding the outliers *Wolbachia *and *Dehalococcoides ethenogenes 195*, as discussed in the main text) results in the new parameter values *a *= 18.26 and *b *= 3650 for Eqn 1.

### Species mixture simulations

In order to generate species mixtures, we first randomly picked 60 out of the entire list of completely sequenced cellular genomes, and simulated WGS sequencing for all 60 genomes using read-length bins from 600 to 900, as described above. We then generated 1,000 simulated metagenomes, by repeating the following procedure 1,000 times.

First, a random number *i *of species were picked (with 1 <*i *≤ 60). Second, for each of the *i *species, its contributing nucleotides *n*_*i *_was randomized, using the following condition regarding the total number of nucleotides in the metagenome:

∑inintotal=1     (2)
 MathType@MTEF@5@5@+=feaafiart1ev1aaatCvAUfeBSjuyZL2yd9gzLbvyNv2Caerbhv2BYDwAHbqedmvETj2BSbqee0evGueE0jxyaibaiKI8=vI8tuQ8FMI8Gi=hEeeu0xXdbba9frFj0=OqFfea0dXdd9vqai=hGuQ8kuc9pgc9s8qqaq=dirpe0xb9q8qiLsFr0=vr0=vr0dc8meaabaqaciGacaGaaeqabaqadeqadaaakeaadaaeqbqaamaalaaabaGaamOBamaaBaaaleaacaWGPbaabeaaaOqaaiaad6gadaWgaaWcbaGaamiDaiaad+gacaWG0bGaamyyaiaadYgaaeqaaaaakiabg2da9iaaigdacaWLjaGaaCzcamaabmaabaGaaGOmaaGaayjkaiaawMcaaaWcbaGaamyAaaqab0GaeyyeIuoaaaa@4395@

Third, a readlength was randomly picked from the available 600, 700, 800, or 900 bp. Fourth, a 'large' metagenome was generated (total sequence about 40 *Escherichia coli *K12 sized genomes) by randomly extracting reads from each of the *I *contributing species, using the read length randomly picked in lead three. Fifth, the theoretical genome size *T *of the simulated metagenome is calculated from the actual contributions *c*_*i *_and from the genome sizes *s*_*i *_of the given species, using the following equations:

T=∑icisi=∑inisiusi=∑iniu, with ci=nisiu, and u=∑inisi     (3)
 MathType@MTEF@5@5@+=feaafiart1ev1aaatCvAUfeBSjuyZL2yd9gzLbvyNv2Caerbhv2BYDwAHbqedmvETj2BSbqee0evGueE0jxyaibaiKI8=vI8tuQ8FMI8Gi=hEeeu0xXdbba9frFj0=OqFfea0dXdd9vqai=hGuQ8kuc9pgc9s8qqaq=dirpe0xb9q8qiLsFr0=vr0=vr0dc8meaabaqaciGacaGaaeqabaqadeqadaaakeaacaWGubGaeyypa0ZaaabuaeaacaWGJbWaaSbaaSqaaiaadMgaaeqaaOGaam4CamaaBaaaleaacaWGPbaabeaaaeaacaWGPbaabeqdcqGHris5aOGaeyypa0ZaaabuaeaadaWcaaqaaiaad6gadaWgaaWcbaGaamyAaaqabaaakeaacaWGZbWaaSbaaSqaaiaadMgaaeqaaOGaamyDaaaaaSqaaiaadMgaaeqaniabggHiLdGccaWGZbWaaSbaaSqaaiaadMgaaeqaaOGaeyypa0ZaaabuaeaadaWcaaqaaiaad6gadaWgaaWcbaGaamyAaaqabaaakeaacaWG1baaaaWcbaGaamyAaaqab0GaeyyeIuoakiaacYcacaqGGaGaae4DaiaabMgacaqG0bGaaeiAaiaabccacaWGJbWaaSbaaSqaaiaadMgaaeqaaOGaeyypa0ZaaSaaaeaacaWGUbWaaSbaaSqaaiaadMgaaeqaaaGcbaGaam4CamaaBaaaleaacaWGPbaabeaakiaadwhaaaGaaiilaiaabccacaqGHbGaaeOBaiaabsgacaqGGaGaamyDaiabg2da9maaqafabaWaaSaaaeaacaWGUbWaaSbaaSqaaiaadMgaaeqaaaGcbaGaam4CamaaBaaaleaacaWGPbaabeaaaaaabaGaamyAaaqab0GaeyyeIuoakiaaxMaacaWLjaWaaeWaaeaacaaIZaaacaGLOaGaayzkaaaaaa@6E88@

Sixth, the effective genome size for the simulated metagenome is predicted from the randomly extracted reads, as described in the main text (Eqn1). Seventh (and finally), errors *e *are calculated using the following equation:

e=EGS−TT     (4)
 MathType@MTEF@5@5@+=feaafiart1ev1aaatCvAUfeBSjuyZL2yd9gzLbvyNv2Caerbhv2BYDwAHbqedmvETj2BSbqee0evGueE0jxyaibaiKI8=vI8tuQ8FMI8Gi=hEeeu0xXdbba9frFj0=OqFfea0dXdd9vqai=hGuQ8kuc9pgc9s8qqaq=dirpe0xb9q8qiLsFr0=vr0=vr0dc8meaabaqaciGacaGaaeqabaqadeqadaaakeaacaWGLbGaeyypa0ZaaSaaaeaacaWGfbGaam4raiaadofacqGHsislcaWGubaabaGaamivaaaacaWLjaGaaCzcamaabmaabaGaaGinaaGaayjkaiaawMcaaaaa@3DC5@

Results are given in Additional data file 3.

### Mixed read lengths simulations

We further generated datasets with mixed read lengths. Specifically, we applied the following procedure 1,000 times.

First, a species *S *was randomly picked from the pool of completely sequenced genomes. Second, a random number *j *of read lengths (with 1 <*j *≤ 4) were picked. Third, a 'large' metagenome was randomly generated (total sequence about 40 *Escherichia coli *K12 sized genomes), consisting only of species *S *(with genome size *s*). Fourth, genome size is predicted, as described in the main text (Eqn 1). Fifth, errors *e *are calculated using the following equation:

e=EGS−ss     (5)
 MathType@MTEF@5@5@+=feaafiart1ev1aaatCvAUfeBSjuyZL2yd9gzLbvyNv2Caerbhv2BYDwAHbqedmvETj2BSbqee0evGueE0jxyaibaiKI8=vI8tuQ8FMI8Gi=hEeeu0xXdbba9frFj0=OqFfea0dXdd9vqai=hGuQ8kuc9pgc9s8qqaq=dirpe0xb9q8qiLsFr0=vr0=vr0dc8meaabaqaciGacaGaaeqabaqadeqadaaakeaacaWGLbGaeyypa0ZaaSaaaeaacaWGfbGaam4raiaadofacqGHsislcaWGZbaabaGaam4CaaaacaWLjaGaaCzcamaabmaabaGaaGynaaGaayjkaiaawMcaaaaa@3E04@

Results are given in Additional data file 3.

### Estimation of effective genome size restricted to bacteria

For EGS estimation restricted to only the bacteria in the sample, we calculate a domain-specific marker gene density *x*_bacteria_, by dividing the number of hits to marker gene OGs (*n*) by the number of hits to any OG (*n*_total_), with the limitation that an OG mapping is only counted if the best BLAST hit of that read region to STRING is a bacterial protein. In this way, only reads of bacterial origin are considered. Because marker gene density is now estimated per read rather than per base pair, this measure requires a new calibration analogous to the one described above. Again, we found Eqn 1 to be the best fitting simple formula for simulated data (R^2 ^= 0.93), with parameter estimates *a *= 0.0389, *b *= 0.81, and *c *= 0.78 from a weighted fit to a training dataset consisting of data for half of the included genomes.

Performance was tested on the remaining data as for the general measure (see Additional data file 1 and Additional data files 5 and 6). Comparison with real reads, as above, revealed an average bias of 5.2%, which lead to adjusted parameter values of *a *= 0.0370 and *b *= 0.770. After correction, we found an unsigned median error of 8.0% (SD 14.6%). An analogous procedure was also performed to estimate EGS restricted to archaeal genomes in the sample. However, currently only very few archaea are fully sequenced, and hence there was insufficient data for a full fit and error estimation. To allow at least an approximate analysis of the archaeal fraction in the acid mine drainage sequences (see below), we obtained a rough measure by scaling Eqn 1 with the bacterial parameter set to fit simulated data from the available fully sequenced archaea. This resulted in the parameter estimates *a *= 0.045, *b *= 2.91, and *c *= 0.78 for archaea (R^2 ^= 0.87). Median unsigned error on the simulated data was 14.7% and the SD was 15.8%.

Before comparing bacterial EGS estimates across environments, we first confirmed that there were no significant differences among the three whalefall samples and among the six Sargasso Sea samples (excluding sample 1), by calculating a *z *score and *P *value (Additional data file 1; all pairwise raw *P *> 0.05). We then pooled all whalefall samples, and separately all Sargasso Sea samples, to reduce the total number of comparisons to be made. Statistical significance of differences in EGS was then estimated by calculating *z *score and *P *value in all remaining pairwise comparisons, and correcting the resulting raw *P *values for multiple comparisons [48].

### Environmental sequencing data

The same data were used as in the report by Foerstner and coworkers [32], with the exception of the Sargasso Sea data, where now all samples were used. Reads were trimmed as described above. Additional data file 10 gives an overview of sequence data after trimming.

Scripting, statistical analyses, and parameter estimation was performed using the R environment for statistical computing [45] and perl [49].

## Additional data files

The following additional data are available with the online version of this article. Additional data file 1 contains the supplementary methods. Additional data file 2 shows the error distribution for EGS prediction on simulated reads. Additional data file 3 is a figure showing that EGS predictions work well when analyzing mixtures of different species or read lengths. Additional data file 4 gives estimated genome sizes for available unfinished genomic sequencing project datasets. Additional data file 5 shows the error distribution for EGS prediction on simulated reads, using the bacteria-specific version of the prediction formula. Additional data file 6 shows the error distribution for EGS prediction on real reads, using the bacteria-specific version of the prediction formula. Additional data file 7 lists OG markers. Additional data file 8 shows randomly selected genomes and read lengths used for calibration. Additional data file 9 shows shotgun sequencing projects used to estimate cloning bias. Additional data file 10 gives data statistics for available environmental shotgun sequencing datasets (measured after quality clipping). Additional data file 11 gives the error distribution for EGS prediction on real reads.

## Supplementary Material

Additional data file 1 A description of methods, which are supplementary to the manuscript. Click here for file

Additional data file 2 A figure showing the error distribution for EGS prediction on simulated reads. Click here for file

Additional data file 3 A figure showing that EGS predictions work well when analyzing mixtures of different species or read lengths. Click here for file

Additional data file 4 A table summarizing estimated genome sizes for available unfinished genomic sequencing project datasets. Click here for file

Additional data file 5 A figure showing the error distribution for EGS prediction on simulated reads, using the bacteria-specific version of the prediction formula. Click here for file

Additional data file 6 A figure showing the error distribution for EGS prediction on real reads, using the bacteria-specific version of the prediction formula. Click here for file

Additional data file 7 A table summarizing OG markers. Click here for file

Additional data file 8 A table showing randomly selected genomes and read lengths used for calibration. Click here for file

Additional data file 9 A table summarizing the shotgun sequencing projects used to estimate cloning bias. Click here for file

Additional data file 10 A table summarizing the data statistics for available environmental shotgun sequencing datasets (measured after quality clipping). Click here for file

Additional data file 11 A figure showing the error distribution for EGS prediction on real reads. Click here for file

## Figures and Tables

**Table 1:** Predicted EGS on environmental samples

Sample	EGS (complete sample)	EGS (only bacteria)
AMD	2.11 ± 0.30	3.16 ± 0.46
Soil	6.29 ± 0.91	4.74 ± 0.69
Whalefall 1 ('agzo')	3.42 ± 0.49	3.39 ± 0.49
Whalefall 2 ('ahaa')	4.50 ± 0.65	4.02 ± 0.59
Whalefall 3 ('ahai')	3.35 ± 0.48	3.24 ± 0.47
Sargasso sample 1	3.25 ± 0.47	3.39 ± 0.49
Sargasso sample 2	1.48 ± 0.21	1.46 ± 0.21
Sargasso sample 3	1.68 ± 0.24	1.57 ± 0.23
Sargasso sample 4	1.59 ± 0.23	1.50 ± 0.22
Sargasso sample 5	6.20 ± 0.89	1.71 ± 0.25
Sargasso sample 6	4.04 ± 0.58	1.94 ± 0.28
Sargasso sample 7	1.32 ± 0.19	1.35 ± 0.20

Values are expressed as megabases (Mb) ± standard deviation. EGS, effective genome size.
