# Supplementary material for: Prediction of effective genome size in metagenomic samples
Source: Genome Biol. 2007 Jan 15;8(1):R10. doi: 10.1186/gb-2007-8-1-r10 (PMC1839125; doi:10.1186/gb-2007-8-1-r10)
Supplement: Additional data file 1 — A description of methods, which are supplementary to the manuscript. [file gb-2007-8-1-r10-S1.pdf]

Supplementary Methods

Error estimation

General measure

To get an unbiased estimate of the predictive power of our method, we determined relative errors (prediction minus actual genome size, divided by known genome size) for the 50% of simulated genomes that were not used for fitting. Errors were approximately normally distributed (Shapiro-Wilks test P=0.88; additional Figure 1). Median unsigned error was 5.3%, and standard deviation was 8.7% (additional Figure 1). Relative errors showed no systematic dependence on genome size or read length (additional Figure 1).

Errors in predictions derived from simulated data stem from three sources: remaining biological variation in total marker gene count per genome, inaccuracies of read assignment to marker gene OGs via BLAST, and stochastic errors due to limited sequencing coverage. Due to the stringent choice of marker genes, biological variation in total marker gene count is low (standard deviation $\Delta x_{\mathrm{bio}}/x = 2.3\%$). To estimate the stochastic error due to limited sequencing depth, we assume that at a given 'true' marker gene density x, each randomly chosen read has *a priori* an equal chance $p \propto x$ of belonging to a marker gene OG. The number of actually observed marker genes n then follows a binomial distribution with variance $(\Delta n)^2 = n \times (1 - p) \approx n$. Assuming that all three error contributions are statistically independent, we can add the associated variances, resulting in the predicted standard deviation

$$\left.\frac{\Delta EGS}{EGS}\right|_{simulated} = \left.\frac{\Delta x}{x}\right|_{simulated} = \sqrt{0.033^2 + \frac{1}{n} + \left(\frac{\Delta x_{blast}}{x}\right)^2}$$

We can validate the coverage dependence of this formula, and estimate $\Delta x_{\mathrm{blast}}$, by comparison to the errors observed for the reference genomes at different simulated coverages (fixed read length L=800bp; additional Figure 1), resulting in $\Delta x_{\mathrm{blast}}/x \approx 8.5\%$. Thus, in a typical environmental sample, approximately 2/3 of the variance can be attributed to BLAST, 1/4 to limited sequencing depth, and the remaining 1/12 to biological variation in marker gene count.

After adjustment of the formula to compensate for cloning bias using real shotgun reads (see Methods), the errors associated with the wide range of actual experiments included in this data set give rise to additional variance compared to the simulated data (Figure 2), resulting in a median unsigned error of 7.8% (standard deviation 14.4%). This error is approximately normally distributed (P=0.67 from Shapiro-Wilks test), and is independent of genome size (additional Figure 3). Decomposing the error into fixed and sequencing depth dependent parts results in the estimated standard deviation (SD)

$$\frac{\Delta EGS}{EGS} = \frac{\Delta x}{x} = \sqrt{0.136^2 + \frac{1}{n}}$$

Eq.(3) was used to calculate an approximate *z*-score when comparing predictions from different data sets *a* and *b*,

$$z = \frac{EGS_a - EGS_b}{\sqrt{SD_a^{\,2} + SD_b^{\,2}}}$$

This was converted to an approximate *P*-value assuming a normal distribution of *z*.

Bacterial-specific measure

Errors were determined as above; they were approximately normally distributed (Shapiro-Wilks test P=0.074;), and showed no systematic dependence on genome size or read length (additional Figure 4). Median unsigned error for simulated reads was 8.5%, and standard deviation was 14.3%.

Comparison to real reads as above revealed an average bias of 5.2%, which led to adjusted parameter values of a=0.0370 and b=0.770. After this correction, we found an unsigned median error of 8.0% (standard deviation 14.6%) for real reads (additional Figure 5), and we estimated the sequencing-depth dependent standard deviation as

$$\left.\frac{\Delta EGS}{EGS}\right|_{bacteria} = \left.\frac{\Delta x}{x}\right|_{bacteria} = \sqrt{0.137^2 + \frac{1}{n} + \frac{1}{n_{total}}}$$

EGS estimation for species admixtures

The effective genome size (*EGS*) for a mixture of species is defined as the average genome size, weighted by the fraction of genomes each species contributes to the sequences:

$$EGS = \alpha \times EGS_1 + \beta \times EGS_2,$$

where α and β are the fractions of genomes contributed by two species with effective genome sizes EGS_1 and EGS_2, and $\alpha+\beta=1$. At fixed read length L, we can write the prediction equation for *EGS*, Eq.(1) of the main text, as

$$EGS_1 = \frac{c}{x_1} = c\frac{N_1}{H_1},$$

where we have written out the marker gene density x as the ratio of the number of hits to marker gene OGs, H_1, and the number of base pairs from this species, N_1; an analogous equation holds for species 2. Hence, we can write the total effective genome size as

$$EGS = \alpha \times EGS_1 + \beta \times EGS_2 = c\left(\alpha\frac{N_1}{H_1} + \beta\frac{N_2}{H_2}\right)$$

The marker gene OGs were chosen such that the total number of BLAST hits to them is independent of genome size of any given species, and depends only on the fraction of genomes in the sequenced reads from this species. Thus, we have $H_1=\alpha H$ and $H_2=\beta H$ with the overall number of hits to marker genes H, and hence

$$EGS = c\left(\alpha\frac{N_1}{\alpha H} + \beta\frac{N_2}{\beta H}\right) = c\frac{N_1 + N_2}{H} = c\frac{N}{H} = \frac{c}{x}$$

with the overall marker gene density $x=H/N$. This confirms the intuitive understanding that the total number of genomes present in the sample is estimated by H, regardless of whether these represent one individual species or a species mix; mean EGS is then given by the number of base pairs per sequenced genome, i.e., is proportional to N divided by H (see also additional Figure 2).

Mixing reads of different lengths

Eq.(1) for individual read lengths is – over the range of relevant read lengths – very close to an inverse linear relationship,

$$EGS = \frac{a + b/L}{x},$$

with R^2=99.8% for L in 1-bp steps between 300bp and 1200bp at any given x.

Here, we will show that the above equation can be applied exactly to mixes of two read lengths; this can then be trivially generalized to higher numbers of different read lengths. We conclude that Eq.(1) is then also approximately applicable to mixes of different read lengths, which we confirmed by simulations (See below and additional Figure 2).

When mixing reads of two different lengths L_1 and L_2, the total number of hits to marker gene OGs H is the sum of the hits in each read length class, $H = H_1+H_2$, and hence the marker gene density is

$$x = \frac{H}{N} = \frac{H_1 + H_2}{N},$$

Where $N=N_1+N_2$ is the total number of base pairs analyzed.

We now want to estimate the parameter c, which can *a priori* depend both on H and on L, in

$$EGS = \frac{c}{x}$$

$$\Rightarrow c = EGS \times x = EGS \times \frac{H}{N} = EGS \times \frac{H_1 + H_2}{N}$$

We can estimate the effective genome size individually at each read length,

$$EGS = \frac{a + b/L_1}{x_1} = \frac{a + b/L_2}{x_2}.$$

With $x_1=H_1/N_1$, we can rewrite H_1 as:

$$H_1 = N_1\frac{a + b/L_1}{EGS},$$

and analogous for H_2. Inserting this into the equation for c, we find

$$c = \frac{1}{N}\left(a(N_1 + N_2) + b(N_1/L_1 + N_2/L_2)\right).$$

The number of base pairs N is given by the number of reads R of a given length L times that length, $N=R \times L$, with the total number of reads the sum of reads from each read length, $R=R_1+R_2$. With this (and with $N=N_1+N_2$), we have

$$c = a + b\frac{R_1 + R_2}{N} = a + b\frac{R}{N} = a + \frac{b}{L},$$

and hence the same formula as applied to individual read lengths can be applied to read length admixtures.
