# Supplementary material for: Prediction of effective genome size in metagenomic samples
Source: Genome Biol. 2007 Jan 15;8(1):R10. doi: 10.1186/gb-2007-8-1-r10 (PMC1839125; doi:10.1186/gb-2007-8-1-r10)

Additional Figure 1: Error distribution for EGS prediction on simulated reads. On a test set of species not used for parameter estimation, we defined relative errors as (prediction by Eq.(1) – known genome size) / (known genome size). Errors are approximately normally distributed (a) (Shapiro-Wilks test: *P*=0.88). Errors are approximately independent of read length (b) and genome size (c). The dependence of the standard deviation on the sequencing depth is as expected under a simple binomial model (d). Red dashed lines (b-c) and dots (d) are estimated standard deviations; the blue dotted line in (d) is the expected standard deviation under the binomial model.

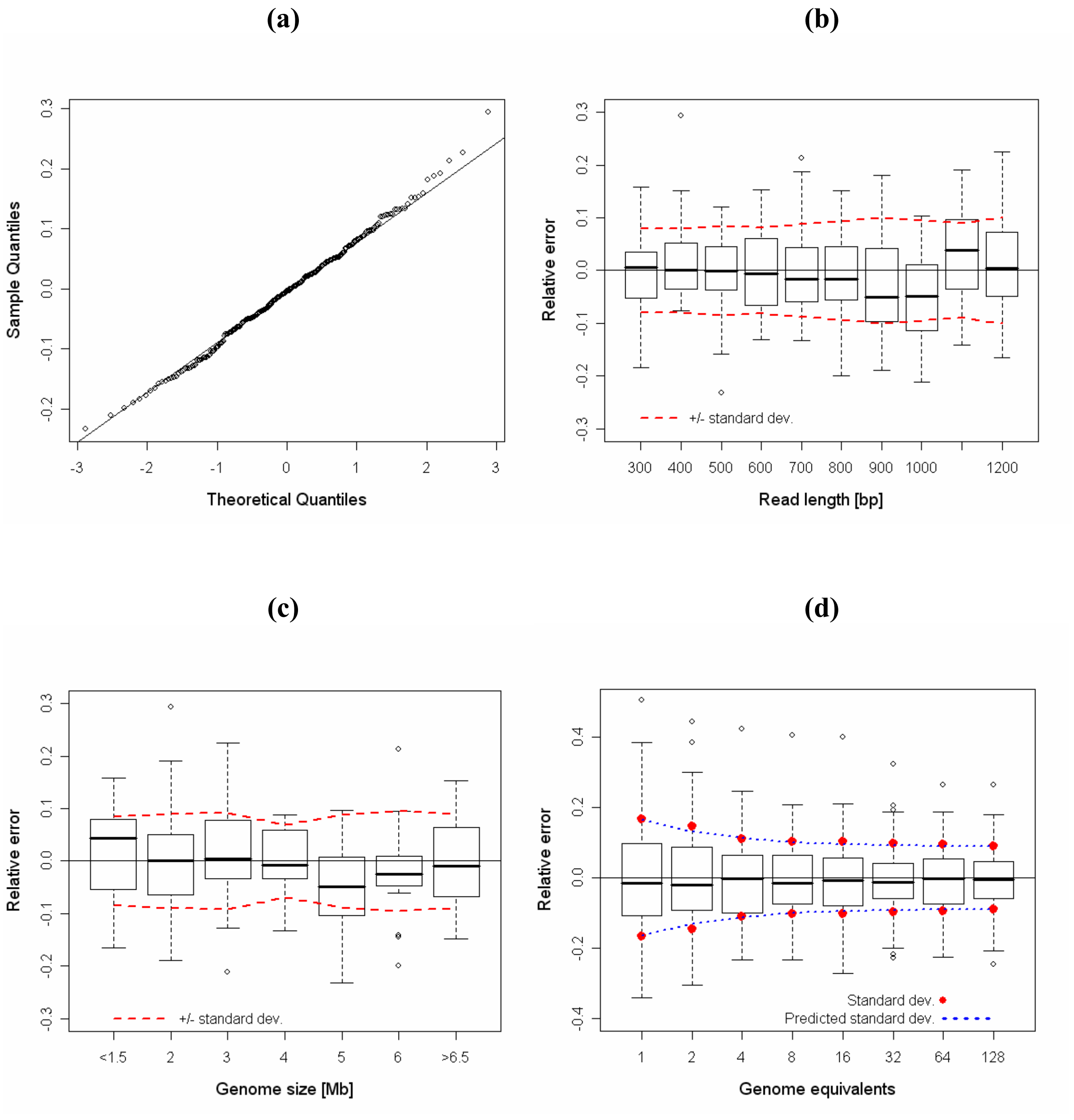

Supplement: Additional data file 2 — A figure showing the error distribution for EGS prediction on simulated reads. [file gb-2007-8-1-r10-S2.pdf]
