# Supplementary material for: Prediction of effective genome size in metagenomic samples
Source: Genome Biol. 2007 Jan 15;8(1):R10. doi: 10.1186/gb-2007-8-1-r10 (PMC1839125; doi:10.1186/gb-2007-8-1-r10)

Additional Figure 2: EGS predictions work well when analyzing mixtures of different species or read lengths. The figure shows the distributions of relative errors (predicted EGS – known EGS) / (known EGS) from genome size predictions with Eq.(1) of the main text, when mixing simulated reads from different species (a) or reads with different read lengths (b). Mixing reads from different genomes or with different read lengths averages out prediction errors due to biological variation and inaccuracies of read assignment to marker gene OGs. Accordingly, the actual standard deviations of the error distributions are substantially lower than the errors predicted for single genomes or readlengths, SD=3.7% (a) and SD=6.4% (b), respectively. See Supplementary Methods for details.

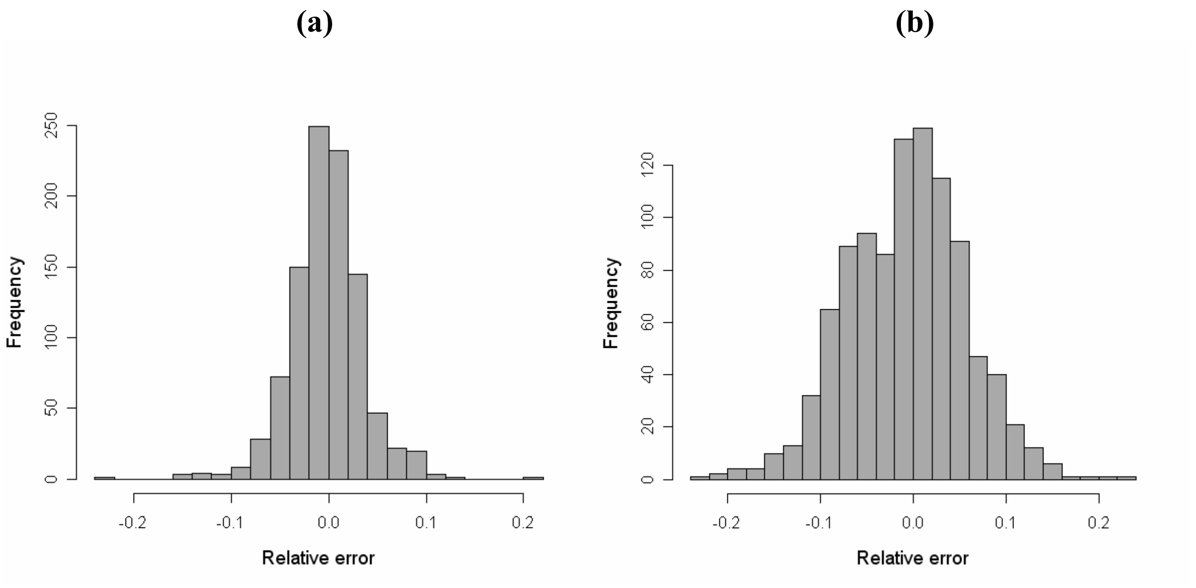

Supplement: Additional data file 3 — A figure showing that EGS predictions work well when analyzing mixtures of different species or read lengths. [file gb-2007-8-1-r10-S3.pdf]
