# Supplementary material for: Prediction of effective genome size in metagenomic samples
Source: Genome Biol. 2007 Jan 15;8(1):R10. doi: 10.1186/gb-2007-8-1-r10 (PMC1839125; doi:10.1186/gb-2007-8-1-r10)
Supplement: Additional data file 4 — A table summarizing estimated genome sizes for available unfinished genomic sequencing project datasets. [file gb-2007-8-1-r10-S4.pdf]

Additional Table 4: Estimated genome sizes for available unfinished genomic sequencing project datasets.

We downloaded and clipped (see Methods) available trace data for ongoing sequencing projects (as listed on the NCBI Microbial Genome Sequencing Projects page; http://www.ncbi.nlm.nih.gov/genomes/lproks.cgi), from the NCBI and Ensembl trace repositories (ftp://ftp.ncbi.nih.gov/pub/TraceDB/; ftp://ftp.ensembl.org/traces/), focusing on unpublished, older and possibly problematic projects, and estimated EGS values for these. On the whole, many of the projects seem to be reasonably close to finishing. Note that our method estimates EGS, which is not reflected in the assembled contigs, so results should be interpreted with data about plasmid numbers in mind, which are unfortunately not to our disposition. Some estimates are, however, extremely high compared to what has been assembled: the *Chloroflexus aurantiacus* genome is predicted to be almost three times larger than what has been assembled up to now. In fact, this estimate might provide an explanation why this genome project has remained unfinished up till now: as the average genome size of currently sequenced chloroflexi is 1.43 Mb, the amount of sequence data needed to finish the assembly could have been seriously underestimated.

				Literature estimates		
**Genome project**	**Predicted genome size**	**Assembled size of major contigs**	**% difference with assembled size**	**Fogel et al. (1999) Microb. Ecol 38:93-113**	**Islas et al., (2004) Orig Life Evol Biosph 34 :243-256**	**other literature**
Azotobacter vinelandii	4,461,110	5.33	16.30%	4.24	4.68	4.70 (1)
Campylobacter lari rm2100	1,589,029	1.56	1.86%	1.84 (Average *Campylobacter*)	1.50 (Average *C.laridis*)	-
Chloroflexus aurantiacus	7,463,901	3.00	148.80%	-	-	-
Desulfitobacterium hafniense	6,755,242	4.90	37.86%	-	-	-
Ferroplasma acidarmanus	1,587,670	1.80	11.80%	-	-	-
Lactobacillus gasseri	1,751,664	1.90	7.81%	2.02	2.02	2.02
Leuconostoc mesenteroides	1,892,346	2.00	5.38%	1.68 (*L. oenos*)	-	-
Novosphingobium aromaticivorans	5,078,347	4.20	20.91%	-	-	-
Oenococcus oeni	3,236,522	1.80	79.81%	-	1.85	1.80 (2)
Streptococcus thermophilus	1,700,028	1.80	5.55%	1.94	1.85	-

(1) Maldonado et al. (1994) J Bacteriol. 176: 3911–3919.

(2) Ze-Ze et al. (2000) Microbiol. 146: 3195-3204.
