# Supplementary material for: Prediction of effective genome size in metagenomic samples
Source: Genome Biol. 2007 Jan 15;8(1):R10. doi: 10.1186/gb-2007-8-1-r10 (PMC1839125; doi:10.1186/gb-2007-8-1-r10)

Additional Figure 4: Error distribution for EGS prediction on simulated reads, using the bacterial-specific version of the prediction formula. On a test set of species not used for parameter estimation, we defined relative errors as (prediction by Eq.(1) – known genome size) / (known genome size). Errors are approximately normally distributed (a) (Shapiro-Wilks test: *P*=0.074). Errors are approximately independent of read length (b) and genome size (c). Red dashed lines (b-c) are estimated standard deviations.

(a)

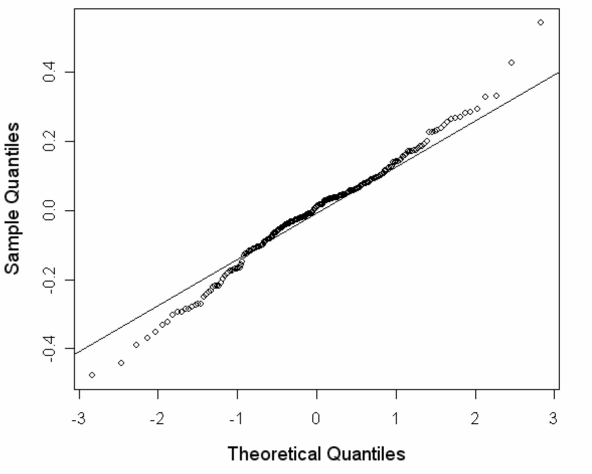

(b)

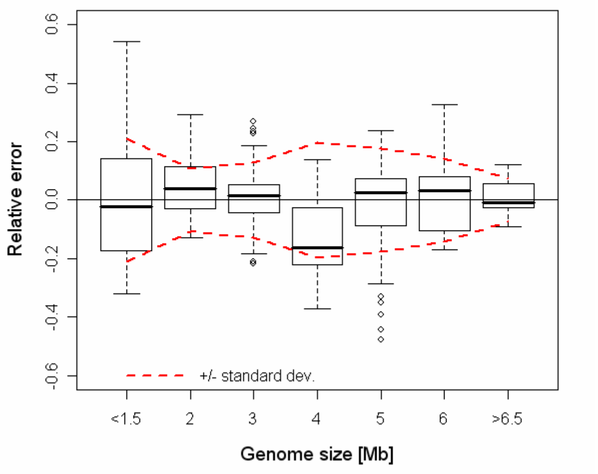

(c)

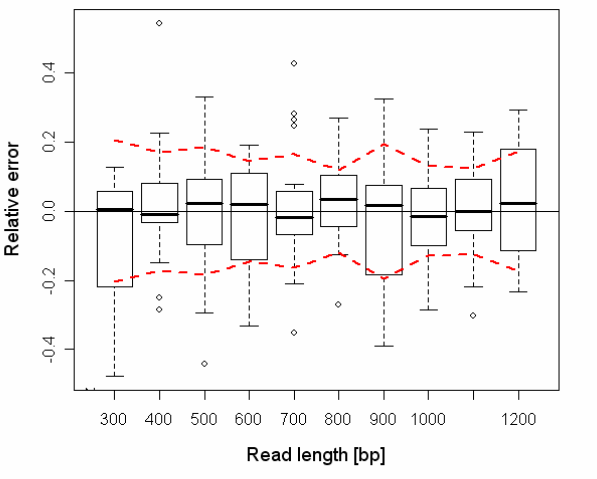

Supplement: Additional data file 5 — A figure showing the error distribution for EGS prediction on simulated reads, using the bacteria-specific version of the prediction formula. [file gb-2007-8-1-r10-S5.pdf]
