# Supplementary material for: Prediction of effective genome size in metagenomic samples
Source: Genome Biol. 2007 Jan 15;8(1):R10. doi: 10.1186/gb-2007-8-1-r10 (PMC1839125; doi:10.1186/gb-2007-8-1-r10)
Supplement: Additional data file 7 — A table summarizing OG markers. [file gb-2007-8-1-r10-S7.pdf]

Additional Table 1: List of orthologous group markers

COG0012 Predicted GTPase, probable translation factor

COG0016 Phenylalanyl-tRNA synthetase alpha subunit

COG0048 Ribosomal protein S12

COG0049 Ribosomal protein S7

COG0052 Ribosomal protein S2

COG0080 Ribosomal protein L11

COG0081 Ribosomal protein L1

COG0085 DNA-directed RNA polymerase, beta subunit/140 kD subunit

COG0087 Ribosomal protein L3

COG0088 Ribosomal protein L4

COG0090 Ribosomal protein L2

COG0091 Ribosomal protein L22

COG0092 Ribosomal protein S3

COG0093 Ribosomal protein L14

COG0094 Ribosomal protein L5

COG0096 Ribosomal protein S8

COG0097 Ribosomal protein L6P/L9E

COG0098 Ribosomal protein S5

COG0099 Ribosomal protein S13

COG0100 Ribosomal protein S11

COG0102 Ribosomal protein L13

COG0103 Ribosomal protein S9

COG0124 Histidyl-tRNA synthetase

COG0184 Ribosomal protein S15P/S13E

COG0185 Ribosomal protein S19

COG0186 Ribosomal protein S17

COG0197 Ribosomal protein L16/L10E

COG0200 Ribosomal protein L15

COG0201 Preprotein translocase subunit SecY

COG0256 Ribosomal protein L18

COG0495 Leucyl-tRNA synthetase

COG0522 Ribosomal protein S4 and related proteins

COG0525 Valyl-tRNA synthetase

COG0533 Metal-dependent proteases with possible chaperone activity

COG0541 Signal recognition particle GTPase
