# Supplementary material for: Prediction of effective genome size in metagenomic samples
Source: Genome Biol. 2007 Jan 15;8(1):R10. doi: 10.1186/gb-2007-8-1-r10 (PMC1839125; doi:10.1186/gb-2007-8-1-r10)
Supplement: Additional data file 8 — A table showing randomly selected genomes and read lengths used for calibration. [file gb-2007-8-1-r10-S8.pdf]

Additional Table 2: Randomly selected genomes and read lengths used for calibration

Species	Readlength sizes used
Aeropyrum pernix	400, 500, 600, 700, 800
Agrobacterium tumefaciens Cereon	400, 500, 900, 1000, 1200
Agrobacterium tumefaciens Dupont	700, 1000, 1100, 1200
Aquifex aeolicus	400, 500, 900, 1100
Archaeoglobus fulgidus	500, 1100
Bacillus anthracis Ames	300, 800, 900, 1000
Bacillus cereus ATCC 10987	300, 900, 1200
Bacillus cereus ATCC 14579	300, 500, 600
Bacillus halodurans	500, 600, 1000, 1100
Bacillus subtilis	600
Bacteroides thetaiotaomicron	600, 800
Bdellovibrio bacteriovorus	300, 800, 900, 1100, 1200
Bifidobacterium longum	1000, 1100
Blochmannia floridanus	600, 1000
Bordetella bronchiseptica	300, 1000
Bordetella parapertussis	500, 600, 700, 900, 1200
Bordetella pertussis	500, 1100, 1200
Borrelia burgdorferi	300, 800, 1200
Bradyrhizobium japonicum	700, 900, 1200
Brucella melitensis	300, 700, 1000
Brucella suis	400, 500, 600, 700
Buchnera aphidicola APS	400, 1200
Buchnera aphidicola Bp	300, 400, 600
Buchnera aphidicola Sg	400, 500, 900, 1000
Campylobacter jejuni	900, 1100, 1200
Chlamydia muridarum	700, 1000
Chlamydia pneumoniae AR39	900, 1000, 1100, 1200
Chlamydia pneumoniae CWL029	400, 700, 1000
Chlamydia pneumoniae J138	300, 600, 800, 1200
Chlamydia trachomatis	300, 600, 800
Chlamydophila caviae	400, 700, 800
Chlamydophila pneumoniae TW183	400, 800, 900, 1200
Chlorobium tepidum	300, 500, 600, 700, 900
Chromobacterium violaceum	300, 600, 1000
Clostridium acetobutylicum	400, 600, 700
Clostridium perfringens	500, 800, 900, 1000
Clostridium tetani	500, 600, 700, 1000
Corynebacterium glutamicum 13032	400, 600, 700, 1000, 1100
Coxiella burnetii	1100
Deinococcus radiodurans	800, 900, 1100, 1200
Desulfovibrio vulgaris	700, 900, 1100
Enterococcus faecalis	800
Escherichia coli EDL933	700, 800
Escherichia coli K12	300, 800
Escherichia coli O157:H7	900, 1000

Escherichia coli O6	400, 500, 700, 1000, 1100, 1200
Fusobacterium nucleatum	700, 1100, 1200
Geobacter sulfurreducens	1100
Gloeobacter violaceus	300, 400, 800, 900
Haemophilus ducreyi	1200
Haemophilus influenzae	1100
Halobacterium sp. NRC-1	700, 900
Helicobacter hepaticus	400, 500, 700, 1100
Helicobacter pylori 26695	500, 800, 900, 1100, 1200
Helicobacter pylori J99	400
Lactobacillus johnsonii	600, 1100
Lactobacillus plantarum	400, 1200
Lactococcus lactis	1000
Leptospira interrogans 56601	700, 900
Leptospira interrogans L1-130	300, 500, 600, 1000
Listeria innocua	500, 600, 1100
Listeria monocytogenes EGD	400, 500, 800, 1200
Listeria monocytogenes F2365	600, 1100
Mesorhizobium loti	600, 1000
Methanobacterium thermautotrophicum	300, 400, 1200
Methanococcus jannaschii	500, 600, 800, 900, 1200
Methanopyrus kandleri	300, 400, 600, 700, 1000, 1100, 1200
Methanosarcina acetivorans	300, 800
Methanosarcina mazei	900, 1000
Mycobacterium bovis	400, 600, 800, 1100
Mycobacterium leprae	300
Mycobacterium tuberculosis CDC1551	300, 700, 900, 1000, 1100, 1200
Mycobacterium tuberculosis H37Rv	700, 1100, 1200
Mycoplasma gallisepticum	300, 1000, 1100
Mycoplasma genitalium	500, 600, 800, 1100
Mycoplasma mobile	300, 500, 800
Mycoplasma mycoides	300, 400, 800, 900, 1200
Mycoplasma penetrans	400, 500, 1200
Mycoplasma pneumoniae	300, 900, 1000, 1200
Mycoplasma pulmonis	500, 1200
Nanoarchaeum equitans	500, 600, 800, 1000
Neisseria meningitidis A	400, 700, 800, 1000, 1100
Neisseria meningitidis B	800, 900, 1100
Nitrosomonas europaea	800, 1200
Nostoc sp. PCC 7120	300, 700, 1000, 1100, 1200
Oceanobacillus iheyensis	300, 1000, 1100
Pasteurella multocida	500, 600, 800, 900
Photobacterium profundum	1100, 1200
Photorhabdus luminescens	400
Phytoplasma Onion yellows	300, 500, 900, 1100
Porphyromonas gingivalis	700, 800, 900
Prochlorococcus marinus CCMP1378	700, 800, 900
Prochlorococcus marinus MIT9313	600, 800, 900, 1000
Prochlorococcus marinus SS120	400, 1200
Pseudomonas aeruginosa	400, 500, 700, 1200

Pseudomonas putida	300, 400, 500
Pseudomonas syringae	400, 700, 800, 900, 1100
Pyrobaculum aerophilum	1000, 1100
Pyrococcus abyssi	400, 600, 700, 800, 1000, 1200
Pyrococcus furiosus	700, 800, 1200
Pyrococcus horikoshii	300, 900, 1200
Ralstonia solanacearum	300, 400, 500, 1200
Rhodopseudomonas palustris	300, 400, 500, 700, 800, 900, 1200
Rickettsia conorii	400, 800, 1000
Rickettsia prowazekii	300, 400, 1100
Salmonella enterica	300, 400, 800, 1000, 1200
Salmonella typhi	1000, 1100
Salmonella typhimurium	400, 500, 700, 800, 1000
Shewanella oneidensis	300, 400, 500, 600, 700, 1000
Shigella flexneri 2a 2457T	300, 500, 700, 1000, 1200
Sinorhizobium meliloti	300, 400, 700, 800, 900, 1000
Staphylococcus aureus MW2	1000, 1100
Staphylococcus aureus Mu50	400, 500, 700, 900
Staphylococcus aureus N315	300, 500, 1000, 1100, 1200
Staphylococcus epidermidis	500, 600, 700
Streptococcus agalactiae III	600, 900, 1100
Streptococcus agalactiae V	700, 1100
Streptococcus mutans	1000
Streptococcus pneumoniae R6	800, 900, 1200
Streptococcus pneumoniae TIGR4	800, 900, 1100
Streptococcus pyogenes M1	600, 800
Streptococcus pyogenes MGAS315	300, 500, 900
Streptococcus pyogenes MGAS8232	500, 600
Streptococcus pyogenes SSI-1	300, 500, 600, 700, 800, 1000, 1200
Streptomyces avermitilis	600, 1200
Streptomyces coelicolor	500, 600, 1100
Sulfolobus solfataricus	300, 600, 700, 1200
Sulfolobus tokodaii	800, 900
Synechococcus elongatus	300, 500, 800, 1200
Synechococcus sp. WH8102	400, 700, 900
Synechocystis sp. PCC6803	800, 900
Thermoanaerobacter tengcongensis	400, 700, 800, 900
Thermoplasma acidophilum	400, 800, 1100
Thermoplasma volcanium	500, 600, 1200
Thermotoga maritima	600, 900
Thermus thermophilus	600, 1000, 1200
Treponema denticola	300, 400, 500, 600, 700, 800, 900
Treponema pallidum	900
Tropheryma whipplei TW08/27	1000, 1100
Ureaplasma parvum	400, 500
Vibrio cholerae	300, 600, 1100
Vibrio parahaemolyticus	500, 600, 1100
Vibrio vulnificus CMCP6	400, 700
Vibrio vulnificus YJ016	700, 1000, 1100
Wigglesworthia brevipalpis	500, 700, 800, 1100

Wolbachia sp. wMel	300, 400, 700
Wolinella succinogenes	300, 400, 500, 900, 1000
Xanthomonas axonopodis	500
Xanthomonas campestris	600, 900, 1000, 1100, 1200
Xylella fastidiosa 700964	300, 400, 1000, 1200
Xylella fastidiosa 9a5c	400, 500, 600, 700
Yersinia pestis CO92	800, 900
Yersinia pestis KIM	300, 400, 700, 800, 1000
Yersinia pestis Medievalis	300, 900
