# Supplementary material for: Prediction of effective genome size in metagenomic samples
Source: Genome Biol. 2007 Jan 15;8(1):R10. doi: 10.1186/gb-2007-8-1-r10 (PMC1839125; doi:10.1186/gb-2007-8-1-r10)
Supplement: Additional data file 9 — A table summarizing the shotgun sequencing projects used to estimate cloning bias. [file gb-2007-8-1-r10-S9.pdf]

Additional Table 3: Shotgun sequencing projects used to estimate cloning bias. Sequence statistics were measured after trimming.

Species	#nucleotides	#reads	average read length
bacillus anthracis str ames	55959099	114159	490.19
bacillus cereus atcc 10987	110580010	159666	692.57
brucella suis 1330	27154557	37870	717.05
burkholderia mallei atcc 23344	98761376	167050	591.21
campylobacter jejuni rm1221	15394604	24657	624.35
chlamydia muridarum nigg	7547044	18795	401.55
chlamydophila caviae gpic	10754382	19409	554.09
chlorobium tepidum tls	13169968	34005	387.30
colwellia psychrerythraea 34h	44208367	68452	645.83
coxiella burnetii rsa 493	58790922	80520	730.14
dechloromonas aromatica rcb	91503870	131331	696.74
dehalococcoides ethenogenes 195	10345211	24689	419.02
enterococcus faecalis v583	16215162	36840	440.15
geobacter sulfurreducens pca	29082168	56430	515.37
listeria monocytogenes str 4b f2365	38678250	57392	673.93
methylococcus capsulatus str bath	28332293	41703	679.38
mycobacterium tuberculosis cdc1551	15497100	48288	320.93
neisseria meningitidis mc58	13711896	36912	371.48
porphyromonas gingivalis w83	14404514	35612	404.48
prochlorococcus sp natl2a	21825174	32942	662.53
pseudomonas putida kt2440	47943324	124665	384.58
pseudomonas syringae pv syringae b728a	66055337	108648	607.98
pseudomonas syringae pv tomato str dc3000	56468618	80760	699.22
psychrobacter sp 273-4	38868860	65342	594.85
ralstonia eutropha jmp134	89558712	148807	601.84
shewanella oneidensis mr-1	21340384	51648	413.19
staphylococcus aureus col	23768569	54913	432.84
staphylococcus epidermidis rp62a	82559248	120637	684.36
streptococcus agalactiae 2603v r	49536795	70603	701.62
streptococcus pneumoniae tigr4	4712789	11787	399.83
thermobifida fusca yx	74123360	129107	574.12
treponema denticola atcc 35405	27703695	48813	567.55
wolbachia endosymbiont of drosophila melanogaster	20127693	29617	679.60
