# Supplementary material for: Prediction of effective genome size in metagenomic samples
Source: Genome Biol. 2007 Jan 15;8(1):R10. doi: 10.1186/gb-2007-8-1-r10 (PMC1839125; doi:10.1186/gb-2007-8-1-r10)
Supplement: Additional data file 10 — A table summarizing the data statistics for available environmental shotgun sequencing datasets (measured after quality clipping). [file gb-2007-8-1-r10-S10.pdf]

Additional Table 5: Data statistics for available environmental shotgun sequencing datasets (measured after quality clipping)

Sample	total nucleotide count	number of reads	average read length	cell size due to filtering
acid mine drainage	76,728,027	103,605	740.58	no
whalefallAGZO	27,365,693	37,583	728.14	no
whalefallAHAA	23,382,344	36,076	648.14	no
whalefallAHAI	24,516,126	36,423	673.09	no
soil	98,442,789	145,503	676.57	no
sargasso sample1	554,785,149	644,320	861.04	0.1-0.8 µm
sargasso sample2	272,963,119	316,776	861.69	0.22-0.8 µm
sargasso sample3	315,627,830	368,694	856.07	0.22-0.8 µm
sargasso sample4	290,599,003	332,014	875.26	0.22-0.8 µm
sargasso sample5	118,650,701	141,001	841.49	3.0-20.0 µm
sargasso sample6	78,220,704	90,867	860.83	0.8-3.0 µm
sargasso sample7	82,521,289	92,327	893.79	0.1-0.8 µm
