# Supplementary material for: Prediction of effective genome size in metagenomic samples
Source: Genome Biol. 2007 Jan 15;8(1):R10. doi: 10.1186/gb-2007-8-1-r10 (PMC1839125; doi:10.1186/gb-2007-8-1-r10)

Figure 3: Error distribution for EGS prediction on real reads. Relative errors are approximately normally distributed (a) (Shapiro-Wilks test: *P*=0.67), and are independent of genome size (b). The red circles in (b) represent two data sets suffering from strong experimental biases (see main text).

(a)

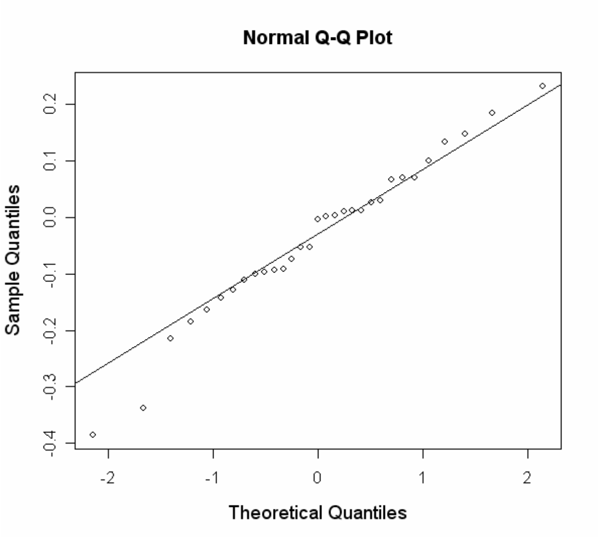

(b)

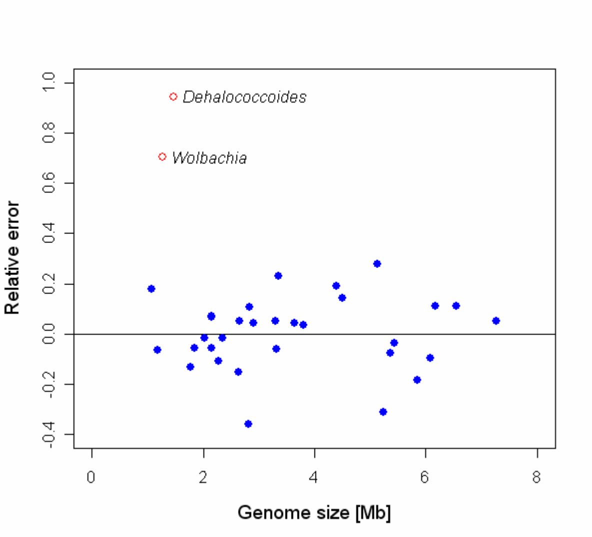

Supplement: Additional data file 11 — A figure showing the error distribution for EGS prediction on real reads. [file gb-2007-8-1-r10-S11.pdf]
